# Quarantine on the Rio Grande

**Published:** 1885-09

**Authors:** 


### QUARANTINE ON THE RIO GRANDE.

"In time of peace prepare for war," is the motto of modern sanitarians. Yellow fever being present in Vera Cruz and other Mexican cities, and free and unrestrained intercourse being daily between that country and Texas, in violation of quarantine proclamation No. 1, of March 20, by the Texas Health authorities, the Governor has issued a second proclamation, establishing rigid quarantine all along the Rio Grande, taking effect August 13. The State Health Officer has instituted a system of railroad inspection of all incoming trains from Mexico, and has also established Inspectors at all the gates of commerce and travel on the border—El Paso, Laredo and Brownsville. Passengers on trains coming east from the Rio Grande will be required to show passports from the officers at El Paso and Laredo that they have not within a period of twenty days, been in Vera Cruz, or any other place infected with yellow fever. The penalty for violation of this proclamation will be fine and imprisonment. Health Officer Swearingen, ever on the alert, paid a visit to Matamoras on 20th August last.

